# Multidisciplinary care meeting practices across diverse international settings

**DOI:** 10.1002/cam4.70136

**Published:** 2024-08-21

**Authors:** Alisha R. Pershad, Dylan Graetz, Mai An Le, Heather Forrest, Miriam Gonzalez‐Guzman, Paola Friedrich

**Affiliations:** ^1^ School of Medicine and Health Sciences The George Washington University Washington District of Columbia USA; ^2^ Department of Global Pediatric Medicine St Jude Children's Research Hospital Memphis Tennessee USA; ^3^ Rhodes College Memphis Tennessee USA

**Keywords:** communication, global health, multidisciplinary care, pediatric cancer

## Abstract

**Purpose:**

Multidisciplinary care (MDC) meetings improve the quality of cancer care by providing a space for interdisciplinary communication. The Pediatric Oncology Facility Integrated Local Evaluation (PrOFILE) tool assesses MDC meetings as part of the Service Integration module. We aimed to evaluate the characteristics of MDC meetings at institutions that completed PrOFILE.

**Methods:**

From 2019 to 2021, 112 institutions from 23 countries collected data by utilizing the abbreviated version of PrOFILE. Within a secondary data analysis, we descriptively analyzed the characteristics of MDC meetings stratified by income level.

**Results:**

Participating institutions were located in low‐income countries (LICs) (*n* = 6), lower‐middle‐income countries (LMICs) (*n* = 34), upper‐middle‐income countries (UMICs) (*n* = 55), and high‐income countries (HICs) (*n* = 17). Of the 112 participating facilities, 79% reported having MDC meetings. The existence of an MDC varied with income, with 50% of LICs and 100% of HICs hosting MDCs. The frequency of MDC meetings also differed, with 100% of MDCs in LICs occurring weekly, while 53% of MDCs in HICs occurred monthly. Specialties regularly represented at MDC meetings across all participating institutions were hematology/oncology (93%), pathology (52%), radiology (60%), general surgery (57%), and radiation oncology (51%). All MDC meetings in LICs reported representation from these specialties. Availability of test results and discussion of new cases did not vary with income. Residual disparities were identified for the following characteristics: discussion of new and interesting cases, inclusion of patient preferences, and ability to meet urgently.

**Conclusions:**

The existence and components of a functional MDC meeting may vary between countries' income levels. Variation in certain components, such as access to tests, may be due to differences in resource distribution, but other factors such as inclusion of patient preferences and ability to meet urgently can be optimized in all settings to foster high‐quality teamwork and communication.

## 1 INTRODUCTION

Multidisciplinary teamwork advances high‐quality care for children and adolescents with cancer.^1^ The terms *interdisciplinary* and *multidisciplinary* are often used interchangeably; however, they are not synonymous. The term “interdisciplinary” describes the presence of multiple disciplines working together to synthesize and harmonize their efforts in a coordinated way.^2^ The term “multidisciplinary” is more general and refers to any space where knowledge from different disciplines is brought together to collaborate, yet disciplines stay within their boundaries.^2^ An example of multidisciplinary teamwork is a multidisciplinary care (MDC) meeting, which describes any group of healthcare professionals from various disciplines gathered together.

Theoretically, MDC meetings improve the quality of cancer care; they provide a space for collaboration, foster shared decision‐making, and improve consistency in patient management in the face of complex disease processes.^3^ However, there are conflicting results in the literature about whether MDC meetings have a positive effect on the quality of cancer and outcomes in high‐income settings.^4^, ^5^, ^6^, ^7^, ^8^, ^9^, ^10^, ^11^ Despite mixed results, more proximal variables of assessment such as patient satisfaction, service integration, and interprofessional communication indicate that a multidisciplinary approach to cancer care is generally accepted as good practice.^12^

Compared to the available literature on optimizing MDC practices in high‐income settings, there is a paucity in the literature studying MDC in low‐ and middle‐income settings. Previous regional work on pediatric hematology‐oncology (PHO) centers in Central America has shown that improving multidisciplinary teamwork is perceived as a priority and an important component of pediatric oncology care in both lower and higher income countries.^13^, ^14^ Furthermore, the influence of non‐biologic factors, like quality teamwork, is typically more actionable than biologic factors in the delivery of quality cancer care. It is especially important to optimize actionable non‐biologic factors in low‐income settings, because the use of limited resources must be maximized for high‐quality care.^15^

The St. Jude Pediatric Oncology Facility Integrated Local Evaluation (PrOFILE) tool is designed for comprehensive evaluation of PHO health service delivery, including the existing structure, characteristics, and communication practices of MDC meetings in participating institutions.^16^, ^17^, ^18^ The present study aimed to analyze trends in MDC communication practices across institutions that completed the Abbreviated Version of PrOFILE stratified by income level. We stratified our analysis by income level to identify the impact a country's income level has on MDC communication practices and to identify easily implementable areas of improvement that facilities can leverage to improve their MDC meetings, independent of their country's income level.

## 2 MATERIALS AND METHODS

### 2.1 PrOFILE implementation

PrOFILE is a validated self‐assessment tool used internationally to provide a dynamic 360° evaluation of health service delivery and helps participating teams to identify institutional priorities.^16^, ^17^, ^18^ The PrOFILE team respondents consisted of a Site Liaison, a PHO medical director, and a data entry lead at each participating PHO institution. Under the guidance of the St. Jude Global Regional Program team and the PrOFILE leadership team, the data entry lead collects and submits data on the 12 module forms. Teams utilize this data to identify their relative strengths and weaknesses, as well as plan future projects accordingly.

From 2019 to 2021, 112 institutions from 23 countries collected data by utilizing the abbreviated version of PrOFILE. The geographic distribution of participating institutions was classified according to the World Bank Regions.^19^ We conducted a secondary analysis focusing on the characteristics of MDC meetings stratified by country's income level using the World Bank country's income category designations of low‐income, lower‐middle income, upper‐middle income, and high income.^19^ Country's income level served as a proxy for resource level. According to PrOFILE, a MDC meeting was defined as a meeting of a group of professionals from various clinical disciplines who together make decisions about recommended treatment of individual patients; usually this is a meeting to discuss clinical findings, radiologic findings, response to treatment, and make decisions together about next steps in curative or palliative treatment. Pediatric tumor board practices, a type of MDC meeting where interesting or challenging cases are reviewed for educational purposes, were defined in the tool and assessed separately, and were not included in this secondary analysis.

### 2.2 Structure and content of MDC meetings

MDC practices were assessed through 40 of over 300 items included in the tool. The 40‐item service integration form includes questions about the structure and content of the MDC meetings that occur at the institution (Appendix S1). These items were informed by studies focusing on evaluating and improving MDC.^1^, ^20^ To understand the structure of the MDC meetings, their existence, frequency, and specialist attendance were assessed. Teams were asked whether their institution held an MDC meeting, to which the response options were *yes*, *no*, and *do not know*. Teams were asked the frequency of MDC meetings held during the past 12 months. Response options were *weekly*, *twice monthly*, *monthly*, *quarterly*, *less than quarterly*, and *when needed*. Teams were finally asked which specialties attend meetings on a regular basis, and the response options were as follows: *hematology and/or oncology*; *pathology*; *radiology*; *radiation oncology*; *general surgery*; *orthopedics*; *ear*, *nose*, *and throat*; *ophthalmology*; *neurosurgery*; *psychosocial providers*; *palliative care specialists*; *pharmacists*; *dieticians*; *nursing*; *trainees*, *geneticists*; and *other*.

After assessing the structure, teams answered questions about the types of cases presented at their MDC meetings. Teams reported the frequency with which new, interesting, and difficult cases were discussed in a group setting. Response options used a fiveitem Likert scale ranging from *almost never* to *almost always* and *not applicable to my role*.

Teams were asked to evaluate three additional characteristics related to communication practices. Teams responded to how frequently relevant test results, reports, and studies were available during MDC meetings. Second, teams reported how frequently patient preferences were discussed when making decisions in the MDC meetings. Finally, teams reported how frequently their institution's team can be brought together to conduct synchronous multi‐disciplinary case discussion urgently. Response options for these questions used a fiveitem Likert scale ranging from *almost never* to *almost always*.

### 2.3 Improvement strategy prioritization

After submitting their assessment data, each participating institution received a specific descriptive and score‐based PrOFILE report as well as an aggregated report. Using these reports, teams participating in a workshop were asked to report the areas of improvement(s) that they wanted to prioritize in their action plan. The number of institutions reporting that MDC meetings were a priority in their improvement strategy was calculated and stratified by each country's income level.

### 2.4 Data analysis

Participating institutions' characteristics were aggregated and the characteristics of MDC meetings occurring in those institutions were analyzed descriptively using measures of frequency and median calculations. The results were stratified by country's income level by using the Center for Disease Control and Prevention's commercially available *EpiInfo 7* software^21^ and figures were developed using Microsoft PowerPoint. We compared the ability to meet urgently, and the availability of relevant test results and materials across countries' income levels after controlling for the institutions that reported having MDC meetings. The presence of specialist availability at each institution was analyzed in a separate PrOFILE module (Appendix S2). Given institutional differences in specialist availability, we controlled for teams that reported having MDC meetings and availability of specified providers in the frequency analysis for presence of specialists.

## 3 RESULTS

### 3.1 Setting and participants of PrOFILE

**FIGURE 1:**
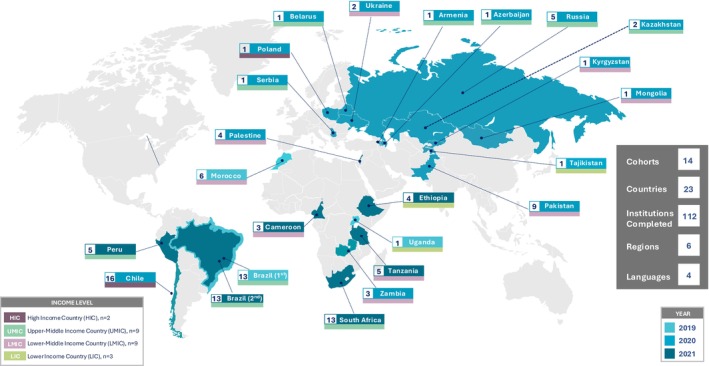
Sites completing the abbreviated version of PrOFILE. This map displays the geographic and income distribution of the 112 institutions participating in PrOFILE across six regions and 23 countries. The four languages represented are English, Spanish, Portuguese, and Russian. The regions are based on the World Bank Regions, a classification used for administrative purposes by the World Bank to assess geographic distribution.^19^ The numbers by each country label indicate the number of institutions participating from that country. The countries are organized by the year that they completed the abbreviation version of PrOFILE, which is defined as a cohort. One cohort of institutions in Brazil completed the tool in 2019, and a second cohort completed the tool in 2020. LIC, low income country, LMIC, lower middle income country, HIC, high income country; UMIC, upper middle income country.

Characteristics of the participating institutions are described in Table 1. The geographic and income distribution of participating institutions is shown in Figure 1. The institutions included were located in low‐income countries (LICs) (5%), lower‐middle‐income (LMICs) (31%), upper‐middle‐income (UMICs) (49%), and high‐income countries (HICs) (15%). Nearly all (98%) participating institutions were PHO hospitals, cancer hospitals or institutes, children's hospitals, or general hospitals. Most hospitals (69%) were public institutions and 66% had at least 15 PHO beds. Most hospitals were also either teaching/training hospitals (90%) or referral hospitals (94%). The number of new cancer cases per year were well‐distributed, with 25% of the participating institutions having fewer than 50 new cases and 41% having more than 100 cases.

**TABLE 1:** Characteristics of participating institutions.

Characteristics	*n* (%)
World bank region, *N* = 112	
East Asia and Pacific	1 (1)
Europe and Central Asia	16 (14)
Latin America and the Caribbean	47 (42)
Middle East and North Africa	10 (9)
North America	‐
South Asia	9 (8)
Sub‐Saharan Africa	29 (26)
Income level, *N* = 112	
Low	6 (5)
Lower middle	34 (31)
Upper middle	55 (49)
High	17 (15)
Type of hospital, *N* = 112	
Pediatric hematology/oncology hospital	13 (12)
Cancer hospital or institute	21 (19)
Children's hospital	25 (22)
General hospital	51 (46)
Other	2 (2)
Type of healthcare system, *N* = 112	
Public	77 (69)
Private, not for profit	23 (21)
Private, for profit	1 (1)
Combination	11 (10)
Government designation, *N* = 112	
Teaching or training hospital	101 (90)
Referral hospital	105 (94)
Pediatric hematology/oncology unit size, *N* = 111	
<15 beds	38 (34)
15–30 beds	40 (36)
>30 beds	33 (30)
New cancer cases per year, *N* = 108	
<50	27 (25)
50–100	37 (34)
>100	44 (41)

Abbreviation: ‐, indicates that no institution responded with a given answer choice.

### 3.2 Structure and content of MDC meetings

Of 112 participating facilities, 79% reported holding MDC meetings. The existence of an MDC meeting varied with country's income; 50% of LICs and 100% of HICs reported holding them. The frequency of MDC meetings also varied. In LICs, 100% of institutions reported that MDC meetings were held weekly, whereas in HICs, only 41% of the institutions reported that MDC meetings were held weekly, while 53% reported that they were held monthly (Table 2). Of the HIC institutions reporting MDC meetings, 41% reported meeting on a weekly basis.

**TABLE 2:** Frequency of multidisciplinary meetings by country's income level.

Characteristics	All participating institutions	Low income	Lower middle income	Upper middle income	High income
	*n* = 112	*n* = 6	*n* = 34	*n* = 55	*n* = 17
Institution holds a MDC meeting, *n* (%)					
Yes	89 (79)	3 (50)	25 (74)	44 (80)	17 (100)
No	23 (21)	3 (50)	9 (26)	11 (20)	‐
Do not know	‐	‐	‐	‐	‐
Frequency of MDC meetings held during the past 12 months, *n* (%)					
Weekly	54 (61)	3 (100)	17 (68)	27 (61)	7 (41)
Twice monthly	11 (12)	‐	2 (8)	8 (18)	1 (6)
Monthly	16 (18)	‐	3 (12)	4 (9)	9 (53)
Quarterly	‐	‐	‐	‐	‐
Less than quarterly	4 (4)	‐	1 (4)	3 (7)	‐
When needed	4 (4)	‐	2 (8)	2 (5)	‐

Abbreviations: MDC, multidisciplinary care; PHO, pediatric hematology/oncology; ‐, indicates that no institution responded with a given answer choice; income level as defined by the World Bank country's income category designations.

After controlling for the institutions that did hold MDC meetings and reported presence of specified providers, specialties regularly represented at these meetings across all income levels consisted of hematology/oncology (93%), pathology (52%), radiology (60%), radiation oncology (51%), and general surgery (57%). Institutions holding MDC meetings in all LICs had reported representation from these specialties. Surgical subspecialists, pharmacists, nurses, and psychosocial support were less frequently represented. Specialties who attended MDC meetings on a regular basis, stratified by country's income level, are shown in Table 3.

**TABLE 3:** Specialist attendance at multidisciplinary meetings by country's income level.

Specialty in attendance	All participating institutions, *n* (%)	Participating institutions by income level, *n* (%)			
		Low income	Lower middle income	Upper middle income	High income
Hematology and/or oncology^1^					
Yes	82 (93)	3 (100)	21 (88)	41 (93)	17 (100)
No	6 (7)	‐	3 (13)	3 (7)	‐
Pathology					
Yes	31 (52)	2 (100)	3 (38)	17 (52)	9 (53)
No	29 (48)	‐	5 (63)	16 (48)	8 (47)
Radiology					
Yes	52 (60)	3 (100)	17 (74)	21 (48)	11 (65)
No	35 (40)	‐	6 (26)	23 (52)	6 (35)
Radiation oncology					
Yes	33 (51)	3 (100)	9 (56)	18 (55)	3 (23)
No	32 (49)	‐	7 (44)	15 (45)	10 (77)
General surgery					
Yes	50 (57)	3 (100)	15 (63)	22 (50)	10 (59)
No	38 (43)	‐	9 (38)	22 (50)	7 (41)
Orthopedics					
Yes	28 (33)	‐	10 (43)	15 (34)	3 (19)
No	58 (67)	3 (100)	13 (57)	29 (66)	13 (81)
Ear, nose and throat					
Yes	12 (15)	‐	6 (25)	5 (13)	1 (7)
No	67 (85)	3 (100)	18 (75)	33 (87)	13 (93)
Ophthalmology					
Yes	18 (22)	1 (33)	10 (43)	6 (15)	1 (6)
No	65 (78)	2 (67)	13 (57)	35 (85)	15 (94)
Neurosurgery					
Yes	30 (36)	1 (33)	9 (43)	16 (36)	4 (27)
No	53 (64)	2 (67)	12 (57)	28 (64)	11 (73)
Palliative care specialist					
Yes	29 (55)	‐	6 (60)	14 (56)	9 (53)
No	24 (45)	1 (100)	4 (40)	11 (44)	8 (47)
Dietician					
Yes	19 (33)	‐	2 (29)	12 (36)	5 (29)
No	38 (67)	‐	5 (71)	21 (64)	12 (71)
Psychosocial providers^2^					
Yes	52 (59)	‐	7 (29)	32 (73)	13 (76)
No	36 (41)	3 (100)	17 (71)	12 (27)	4 (24)

*Note*: Facilities that responded “no” or “do not know” to question 1 of the Service Integration form (Appendix S1) and/or facilities that indicated a specified provider was “not available” in the Personnel form (Appendix S2) were excluded from the analysis.^1^ *n* = 1 (LMIC) indicated that there were no hematology/oncology providers on staff (including fellows), but that hematology/oncology attended the institution's MDC meetings. This institution was excluded from the dataset because conflicting responses were unable to be validated.^2^ Psychosocial provider availability was determined by the presence of a social worker and/or psychologist at the institution.

Abbreviations: MDC, multidisciplinary care; PHO, pediatric hematology/oncology; ‐, indicates that no institution responded with a given answer choice.

**FIGURE 2:**
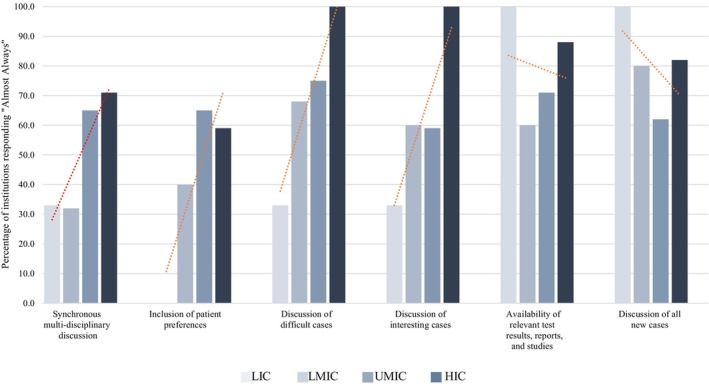
Structure and content of multidisciplinary care meetings by income. A gradient based on income level is observed for the following characteristics: Teams can be brought together to conduct a synchronous multidisciplinary case discussion urgently (*n* = 61), patient preferences are discussed when making decisions (*n* = 40), difficult cases are discussed in group settings (*n* = 77), and interesting cases are discussed in group settings (*n* = 66). A gradient based on income level is not observed for the following characteristics: Relevant test results, reports, and studies are available during the meetings (*n* = 52), and all new cases are discussed in a group setting (*n* = 51). HIC, high‐income country, LIC, low‐income country, LMIC, lower‐middle income country, MDC, multidisciplinary care; UMIC, upper‐middle income country.

At institutions holding MDC meetings, responses to questions about the content and resources of the MDC meetings varied by countries' income level and are shown in Figure 2. Both difficult and interesting cases were “almost always” discussed in 33% of institutions in LICs compared to 100% of MDC meetings in HICs. In 32%–71% of institutions based on countries' income level, teams can be brought together to conduct a synchronous multidisciplinary case discussion urgently. The greatest disparity was observed when looking at whether patient preferences were discussed when making decisions during MDC meetings; no institutions in LICs reported including patient preferences and 62% of institutions in UMICs included them. 100% of institutions in LICs and 88% of those in HICs reported the presence of relevant test results, reports, and studies. 100% of the institutions in LICs and 82% in HICs reported discussing new cases in a group setting. Granular data showing the structure and content of group discussion at participating institutions holding MDC meetings are shown in Appendix S2.

### 3.3 Improvement strategy prioritization

Of the participating institutions, 5 (4%) institutions, all of which were in lower middle‐income countries, reported prioritizing the Service Integration module as part of their improvement strategy. Four of those five institutions specifically reported prioritizing improving their MDC meetings. The majority (54%) of institutions identified priorities relating to patients and outcomes, chemotherapy, diagnostics, and national context.

## 4 DISCUSSION

Our study described the landscape of MDC meetings internationally by income level. Our data suggest that the concept of MDC meetings seems equally adopted in higher and lower income settings, as supported by the fact that these meetings happen at similar or higher frequencies in lower income settings. There also seems to be good adoption of what is considered good practice for these MDC meetings, such as routine scheduling and diverse specialty representation.^1^, ^22^, ^23^ However, we identify residual disparities and opportunities for improvement.

Having relevant test results, reports, and studies available is an important characteristic of MDC meetings.^1^ While it did not show a gradient by income level, this characteristic might be more challenging to target in resource‐limited settings. Previous studies have identified the importance of a representative specialist composition, though it can be challenging to achieve considering the work demands of health professionals.^1^, ^10^ Our results show that core specialties such as hematology/oncology, pathology, radiology, and radiation oncology are well‐represented across income levels; however, LICs and LMICs disproportionately have fewer medical specialists in general, therefore attendance might be a challenging area for improvement.^24^

We also identify characteristics that are important areas for improvement that are not as resource‐dependent, such as the discussion of difficult, new, and interesting cases, ability to meet urgently, and incorporation of patient preferences. It is accepted that the role of MDC meetings is to discuss a diversity of cases.^25^, ^26^, ^27^ MDC meetings allow for the integration of multiple health professionals' opinions and increased incorporation of clinical expertise, which can be integral to addressing the medical complexity of pediatric cancer cases.^25^ Consequently, maximizing the discussion of these types of cases as well as being able to meet urgently to discuss changes to management for these cases is an important improvement opportunity to consider.

Inclusion of patient preferences was the least adopted practice among all institutions, yet it is well accepted that is important to include the patients' opinions and preferences in clinical decision‐making.^28^ While the best way to represent patients' interests in MDC meetings requires further exploration, understanding the goals of patients and their families through shared decision making in the pediatric oncology setting should be standard practice.^29^, ^30^ Accordingly, optimizing communication practices among clinicians to better incorporate those patient values into clinical decision‐making is a key potential area for improvement. Furthermore, many clinicians engaging in MDC experience higher job satisfaction and perceive higher quality care delivery, making this intervention useful for both patients and practitioners.^13^

Few of the institutions that conducted PrOFILE have prioritized improving MDC meetings during their interpretation and action phase and prioritization workshops. While teams were not directly asked why communication practices did not rank highly on their list of priorities, we acknowledge that PrOFILE is a full evaluation tool of health‐system delivery. The modules assess a vast range of priorities, including but not limited to diagnostic tools and treatment practices. Many competing priorities are assessed across the modules; yet some institutions still prioritize addressing MDC meetings. Therefore, we need to evaluate how we support institutions to address their communication practices. Our results demonstrate that opportunities for improvement extend beyond hosting the meetings to boost service integration, communication, patient satisfaction, and interprofessional engagement. Now that we have identified the opportunities for improvement for the participating institutions, further investigation is needed to study how teams are increasing their MDC meetings and adjusting their MDC meetings to address these residual disparities upfront.

The literature describing multidisciplinary communication, especially on a global scale, is limited. Therefore, one important strength of our study is the inclusion of a large, international cohort. Another strength is our collection of the data in a structured way, strengthening the comparisons generated. Despite these strengths, our study has limitations. Although geographic representation was expansive, LICs were less widely represented, limiting the generalizability of our results for institutions in LICs. Furthermore, the 17 institutions from HICs were only from two countries, Poland and Chile, which may not represent trends in communication practices as widely for HICs as LMICs and UMICs. Teams self‐reported their institutions' MDC meeting structure and communication practices, thus potentially leading to recall bias. Finally, this analysis is descriptive in nature, limiting the strength of the conclusions that we can draw, including causality.

Despite these limitations, this analysis is contributing to communication practice improvement internationally by showing teams where they excel and where they can amend their current communication practices independent of their income level. Using the lessons learned from these data, we are currently developing post‐PrOFILE activities, in which teams are exposed to improvement science more broadly and can use those skills to address their communication priorities specifically. Furthermore, St. Jude Global has developed Global Packages so that institutions can implement practice‐based improvement interventions, one of which aims to include communication practices in MDC meetings.

## 5 CONCLUSION

The existence and components of a functional and optimized MDC meeting vary internationally, though generalizability is limited. Differences in some components, such as access to tests, may be due to differences in resource distribution; other factors can be prioritized in all settings, fostering high‐quality teamwork and communication.

## AUTHOR CONTRIBUTIONS

**Alisha R. Pershad:** Writing – original draft (lead). **Dylan Graetz:** Supervision (equal); writing – review and editing (equal). **Mai An Le:** Data curation (equal); formal analysis (equal). **Heather Forrest:** Formal analysis (equal); investigation (equal); supervision (equal). **Miriam Gonzalez‐Guzman:** Conceptualization (equal); methodology (equal); project administration (equal); supervision (equal). **Paola Friedrich:** Conceptualization (lead); methodology (equal); project administration (equal); supervision (equal); writing – review and editing (equal).

## FUNDING INFORMATION

This study was funded, in part, by ALSAC.

## CONFLICT OF INTEREST STATEMENT

The authors have no financial or proprietary interests in any material discussed in this article.

## ETHICS APPROVAL

This secondary analysis is exempt from review by the St. Jude Children's Research Hospital Institutional Review Board.

## PRECIS

The existence and components of a functional and optimized multidisciplinary meeting may vary across income level internationally. The need for certain components may be difficult to uniformly resolve due to differences in resource distribution, but other factors can be optimized in all settings to foster quality teamwork and communication.

## Supporting information


Appendix S1.

Appendix S2.

Appendix S3.

## Data Availability

Available from the authors on request.
