# Supplementary material for: Multidisciplinary care meeting practices across diverse international settings
Source: Cancer Med. 2024 Aug 21;13(16):e70136. doi: 10.1002/cam4.70136 (PMC11336655; doi:10.1002/cam4.70136)
Supplement: Supplementary file 3 — Appendix S3. [file CAM4-13-e70136-s001.docx]

**Appendix 2. Likert Scale Data on Structure and Content of Group Discussion at Institutions Holding Multidisciplinary Care Meetings**

| **Characteristics** | **Median (IQR)**^2^ | **All Participating Institutions,**  n (%) | **Participating Institutions by Income Level,** n (%) | | | |
| --- | --- | --- | --- | --- | --- | --- |
|  |  |  | Low Income, n=3 | Lower Middle Income, n=25 | Upper Middle Income, n=44 | High Income, n=17 |
| Team can be brought together to conduct synchronous multi-disciplinary case discussion (urgent) | 1 (1-2) |  |  |  |  |  |
| *Almost always* |  | 51 (57) | 2 (33) | 11 (32) | 36 (65) | 12 (71) |
| *Frequently* |  | 22 (25) | 0 (0) | 11 (32) | 8 (15) | 5 (29) |
| *Sometimes* |  | 12 (13) | 1 (17) | 7(21) | 8 (15) | 0 (0) |
| *Infrequently* |  | 1 (1) | 0 (0) | 2 (6) | 1 (2) | 0 (0) |
| *Almost never* |  | 3 (3) | 2 (33) | 3 (9) | 2 (4) | 0 (0) |
| *Not applicable to my role* |  | 0 (0) | 0 (0) | 0 (0) | 0 (0) | 0 (0) |
| Patient preferences are discussed when making decisions in the MDC meetings^1^ | 1 (1-2) |  |  |  |  |  |
| *Almost always* |  | 36 (57) | 0 (0) | 4 (40) | 22 (65) | 10 (59) |
| *Frequently* |  | 20 (32) | 2 (100) | 2 (20) | 12 (35) | 4 (24) |
| *Sometimes* |  | 7(11) | 0 (0) | 4 (40) | 0 (0) | 3 (18) |
| *Infrequently* |  | 0 (0) | 0 (0) | 0 (0) | 0 (0) | 0 (0) |
| *Almost never* |  | 0 (0) | 0 (0) | 0 (0) | 0 (0) | 0 (0) |
| *Not applicable to my role* |  | 0 (0) | 0 (0) | 0 (0) | 0 (0) | 0 (0) |
| Relevant test results, reports, and studies are available during MDC meetings^1^ | 1 (1-2) |  |  |  |  |  |
| *Almost always* |  | 47 (75) | 2 (100) | 6 (60) | 24 (71) | 15 (88) |
| *Frequently* |  | 9 (14) | 0 (0) | 2 (20) | 5 (15) | 2 (12) |
| *Sometimes* |  | 4 (6) | 0(0) | 1 (10) | 3 (9) | 0 (0) |
| *Infrequently* |  | 0 (0) | 0 (0) | 0 (0) | 0 (0) | 0 (0) |
| *Almost never* |  | 1 (2) | 0 (0) | 0 (0) | 1 (3) | 0 (0) |
| *Not applicable to my role* |  | 2 (3) | 0 (0) | 1 (10) | 1(3) | 0 (0) |
| Difficult cases are discussed in a group setting | 1 (1-1) |  |  |  |  |  |
| *Almost always* |  | 68 (76) | 1 (33) | 17 (68) | 33 (75) | 17 (100) |
| *Frequently* |  | 16 (18) | 1 (33) | 6 (24) | 9 (20) | 0 (0) |
| *Sometimes* |  | 5 (6) | 1 (33) | 2(8) | 2 (5) | 0 (0) |
| *Infrequently* |  | 0 (0) | 0 (0) | 0 (0) | 0 (0) | 0 (0) |
| *Almost never* |  | 0 (0) | 0 (0) | 0 (0) | 0 (0) | 0 (0) |
| *Not applicable to my role* |  | 0 (0) | 0 (0) | 0 (0) | 0 (0) | 0 (0) |
| Interesting cases are discussed in a group setting | 1 (1-2) |  |  |  |  |  |
| *Almost always* |  | 59 (66) | 1 (33) | 15 (60) | 26 (59) | 17 (100) |
| *Frequently* |  | 19 (21) | 1 (33) | 5 (20) | 13 (30) | 0 (0) |
| *Sometimes* |  | 11 (12) | 1 (33) | 5 (20) | 5 (11) | 0 (0) |
| *Infrequently* |  | 0 (0) | 0 (0) | 0 (0) | 0 (0) | 0 (0) |
| *Almost never* |  | 0 (0) | 0 (0) | 0 (0) | 0 (0) | 0 (0) |
| *Not applicable to my role* |  | 0 (0) | 0 (0) | 0 (0) | 0 (0) | 0 (0) |
| All new cases are discussed in a group setting^1^ | 1 (1-2) |  |  |  |  |  |
| *Almost always* |  | 45 (71) | 2 (100) | 8 (80) | 21 (62) | 14 (82) |
| *Frequently* |  | 12 (19) | 0 (0) | 0 (0) | 9 (26) | 3 (18) |
| *Sometimes* |  | 5 (8) | 0 (0) | 2 (20) | 3 (9) | 0 (0) |
| *Infrequently* |  | 1 (2) | 0 (0) | 0(0) | 1(3) | 0 (0) |
| *Almost never* |  | 0 (0) | 0 (0) | 0 (0) | 0 (0) | 0 (0) |
| *Not applicable to my role* |  | 0 (0) | 0 (0) | 0 (0) | 0 (0) | 0 (0) |

^1^n=63 for” Patient preferences are discussed when making decisions in the MDC meetings”, “Relevant test results, reports, and studies are available during MDC meetings” and “All new cases are discussed in a group setting” because questions were added later. ^2^ Median value calculated using the following Likert scale legend: 1-Almost Always, 2-Frequently, 3-Sometimes, 4-Infrequently, 5-Almost Never, 6-Not applicable for my role. Abbreviations: MDC = multidisciplinary care; IQR = interquartile range
